# The Fungal Mycobiome and Its Interaction with Gut Bacteria in the Host

**DOI:** 10.3390/ijms18020330

**Published:** 2017-02-04

**Authors:** Qi Hui Sam, Matthew Wook Chang, Louis Yi Ann Chai

**Affiliations:** 1 Division of Infectious Diseases, University Medicine Cluster, National University Health System, Singapore 119228, Singapore; qi_hui_sam@nuhs.edu.sg; 2 Department of Biochemistry, Yong Loo Lin School of Medicine, National University of Singapore, 14 Medical Drive, Singapore 117599, Singapore; bchcmw@nus.edu.sg; 3 NUS Synthetic Biology for Clinical and Technological Innovation (SynCTI), Life Sciences Institute, National University of Singapore, 28 Medical Drive, Singapore 117456, Singapore; 4 Yong Loo Lin School of Medicine, National University of Singapore, NUHS Tower Block, Level 11, 1E Kent Ridge Road, Singapore 119228, Singapore

**Keywords:** fungus, mycobiome, gut microbiome, gastrointestinal, microflora, pathobiont

## Abstract

The advent of sequencing technology has endowed us with the capacity to study microbes constituting the human commensal community that were previously non-culturable. Much of the initial works have concentrated on the bacterial flora constituting the gut microbiome, since specimens are readily accessible in health and disease. Less, however, is understood of the “silent population”—the fungal species, also known as the mycobiome. Living in symbiosis with bacteria as commensals in our body, it is perceivable that the mycobiome exerts an inadvertent influence on the microbiome. We review here the recent knowledge gained from study of the interaction between the mycobiome and microbiome in health and disease susceptibility, immunity, and consequences from antimicrobial treatment.

## 1. Introduction

Humans have been interested in the microbial flora found on and in our bodies since the advent of microbiology. In 1681, Antony van Leeuwenhoek, one of the pioneers in the field observed “great numbers of small animals” in his stool. He also looked at the difference in the stool samples when he was well and when he had diarrhoea (reviewed in [1]). Fast forward to modern times, the use of stool samples for gut microbiota studies remains unchanged, and the quest to understand more about the “small animals” with new analysis methods has unveiled many more questions that need to be answered.

The microbiome, also known as the microflora or microbiota, is usually assumed to be the bacterial microbiota. Though bacterial flora on the skin, oral tract, vagina, etc., have been sequenced, the gut flora is the most studied by far [2]. The gut microbiome has been the major topic of interest, due to the large amounts of bacteria found in our gastrointestinal (GI) tract. The National Institutes of Health (NIH) human microbiome project (HMP) and Metagenomics of the Human Intestinal Tract (MetaHIT) projects were initiated to characterize the microbial, namely bacterial microbiota, communities in humans and their impact on human health [3,4]. However, there are other inhabitants of the microbiome too, namely the commensal fungi and archaea. The term “mycobiome”, used to differentiate the mycology aspect of the microbiome, has been gaining momentum—from being first coined in 2009 [5], to 10 results using a Pubmed search in 2013 [6], to at least 70 results as of July 2016.

Commensal fungi that normally inhabit our bodies have been much less studied, as fungi form the minority of the total commensal organisms in humans [7]. A large portion of these fungi are also unculturable [7]. The fungal mycobiome studies, therefore, are important in characterizing these fungi and pave a way for the “good fungi” to be studied. Also, many pathogenic fungi are “pathobionts”—commensals in our bodies that cause no harm in normal conditions, but have pathogenic potential. For example, *Candida albicans*, which causes systemic candidiasis in immunocompromised patients, is a normal part of the gut microflora [8]. What makes these fungi, which are non-pathogenic in normal conditions, turn pathogenic is still a hot topic of debate. The burden of fungal disease forms a substantial amount of the total infectious diseases spectrum. A large proportion consists of fungal infections in immunocompromised patients with an estimated mortality between 35% to 45% (reviewed in [9]).

Currently, the gut microbiome has been linked to many gut-linked conditions, such as inflammatory bowel disease (IBD) and obesity, as well as many seemingly unrelated conditions, such as maturation of the immune system and even cardiac size [2]. However, the role of the mycobiome is still unclear for many of them. Mycobiome studies pose similar, if not more, challenges as microbiome studies. As the microbiome changes in accordance to a person’s age, diet, use of antibiotics, etc., [10,11,12] we can assume the mycobiome changes accordingly too. Researchers are now increasingly aware of the role of the mycobiota in health and disease. Before the development of genomic-based, culture-independent techniques, the gut microflora was mostly studied qualitatively by microscopy and quantitively by anaerobic culture techniques [13,14]. Current methods for microbiome and mycobiome studies are mainly focused on targeted or metagenomic sequencing. The falling costs of sequencing due to next generation sequencing technologies has allowed a much larger set of the microbiome to be sequenced, including the unculturable bacteria, fungi, and archaea [15]. While culture-based techniques have helped identify hundreds of bacterial species in the gut [13,14], next generation sequencing technologies have ballooned the bacterial diversity to over 1000 species [4]. Using classical culture methods before the advent of sequencing, fungal *Candida* species was mostly isolated from human stool and colonic samples [16]. Current state-of-the-art technologies, such as next generation sequencing, pyrosequencing, and targeted sequencing, could remove the culturing-bias and reveal many more unculturable fungi in the gut [16].

**Figure 1:**
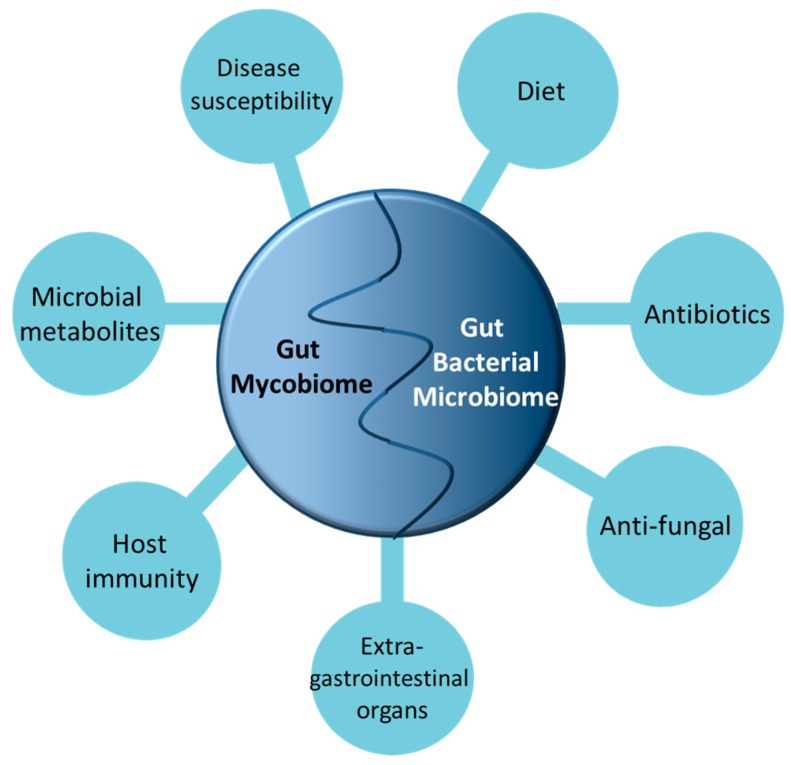
The multifaceted factors influencing gut microbiome and mycobiome interaction.

Mycobiome studies are currently focused on the fungi residing in healthy vs. diseased models, and more research has to go into the interactions between the myco- and microbiomes with the host. In this review, we focus on the role of the fungal mycobiome in their interactions with the gut bacteria and their potential implications in the host (Figure 1).

## 2. Fungi That Are Found in the GI Tract

Reports using next generation sequencing have found diverse fungal communities in all sections of the human gut, consisting mainly of the phyla Ascomycota, Basidiomycota, and Zygomycota [17,18,19]. *Candida* and *Phialemonium* are present in the stomach gastric fluid and are able to survive the low pH [20]. One study found 16 fungal species in stool samples [21], with *Galactomyces geotrichum* being the most prevalent. Another study revealed 66 genera of fungi, with the *Saccharomyces* genus being most prevalent, followed by *Candida* and *Cladosporium* [17], while yet another study found 75 genera of fungi, with *Penicillium*, *Candida*, and *Saccharomyces* being the most prevalent [19]. From the colon mucosa, *Dothideomycete* sp., *G. geotrichum*, and *Ustilago* sp. were the most prevalent [22]. As seen above, a large variety of fungi are found in the human GI tract and there is no consensus yet on the ideal fungal mycobiome—a subject which can be explored in future studies.

There are two general habitats of colonization in the GI tract, the luminal/fecal contents and the mucosa [13]. Mucosa-associated and fecal-associated microbiota have been found to have different microbiota compositions [16]. The mucosal microbiota can be sub-categorized into two layers—those that directly attach to the epithelium and those that are entrapped within the outer mucosal layer, adhering to either the mucus or the primary layer of organisms below it and to each other (reviewed in [14,23]). To study mucosa-associated microbiota, samples have to be taken invasively by biopsies of the intestinal epithelium [22], hence most studies involving the GI mucosa use mouse models, which can be sacrificed to sample their mucosal microbiota. As most human gut micro- and mycobiome studies use the less-invasive way of taking fecal specimens, they may not be fully representative of the GI flora and may contain many microbial organisms which are just “passing through”.

To characterize the mycobiota by sequencing, variable portions of the fungi genome are targeted, which characterize the fungi to genus-level, such as the 18S rDNA or the fungal internal transcribed spacer (ITS) of the rRNA locus (reviewed in [18]). Each sequencing platform has its strengths and weaknesses in terms of read length, cost, and coverage (reviewed in [24,25,26]). The sequences are grouped into Operational Taxonomic Units (OTUs), and the OTUs are compared with existing databases, and the genus abundance is determined from multivariate analysis of the various OTUs. Alternatively, the raw fragment sequences from metagenomics studies are assembled into contigs (continuous sequences) and compared to databases (detailed reviews in [24,25,26]). A variety of multivariate analyses with visualization processes are performed to elucidate the microbial communities. Problems do exist with assigning taxonomy to sequencing reads due to poor annotation of fungal database, misspellings, unfamiliarity with nomenclature, incomplete representation, etc. ([20], reviewed in [18]). The role these unculturable fungi, with unknown taxonomy, play in the gut is not clear, and new methods have to be developed to study their interactions within the gut.

## 3. (Anti)-Fungal and (Anti)-Bacterial Interactions

A classic way to study interaction between the myco- and microbiome is to introduce dysbiosis in the gut through treatment with anti-fungals and anti-bacterials [27,28]. Mouse models have been often used in this way to study the gut microbiota (reviewed in [29,30,31]). Normally, conventional animals, including humans, with intact microbiota are significantly more resistant to pathogenic fungi, namely *C. albicans* colonization compared to animals fed with antibiotics [32]. *C. albicans* is a commensal in the human gut, but not in the murine gut. *Candida* colonization is induced in germ-free, gnotobiotic, infant mice, or conventional mice fed with anti-bacterials and anti-fungals. This gives us an insight into the inter-relations between the gut bacteria and fungi.

Anti-bacterials can work to promote or inhibit pathogenic fungal growth (reviewed in [28]). It has been known for more than 30 years that anti-bacterial treatment, notably broad-spectrum antibiotics and antibiotics specific to anaerobic bacteria, cause differential effects on fungal susceptibility and predisposes patients to gastrointestinal infections by *C. albicans* [14,33,34]. The effect of antibiotics on the bacterial microbiome has been shown in that a treatment course of antibiotics induces changes in composition of the microbiota. In previous studies it was observed that it took at least 40 days from the start of treatment for microbiota to revert back close to their pre-treatment status [35,36]. In human patients, the use of broad-spectrum antibiotics that affect anaerobic bacteria are associated with increased yeast flora in the gut compared with antibiotics with poor anaerobic activity [34]. The impact of antibiotics on the commensal fungal mycobiome is much less known.

Fungi are able to establish a niche in a perturbed gut bacterial microbiome. Studies show that the introduction of small amounts of *C. albicans* to mice after antibiotic treatment caused significant shifts to the gut microbiota, from phyla to family-level, and it may not return to its pre-antibiotic state in the long term [37,38]. Gene-regulation of *Candida* with antibiotics-treated mice also differed from non-treated mice despite absence of intestinal inflammation and absence of *Candida* hyphal invasion [37]. *Enterococcus* sp., mainly *E. faecalis*, was found to predominate *Lactobacillus* following introduction of *C. albicans* into antibiotic-perturbed mouse gut microbiome [38,39]. In a *Caenorhabditis elegans* co-infection model though, *E. faecalis* was found to inhibit *C. albicans* hyphal morphogenesis, possibly by secretion of proteases associated with quorum sensing [40]. *Lactobacillus* and *C. albicans* may compete antagonistically in the murine gut [41]. Clinically, *C. albicans* and *E. faecalis* are frequently co-cultured in nosocomial infections, (reviewed in [42]) which suggests that *E. faecalis* may have adapted mechanisms for permissive growth with *C. albicans*.

Much fewer studies look at the effect of anti-fungal use on the microbiome. A study treating mice with anti-fungals showed decreased fungal diversity but increased bacterial diversity relative to normal control [43]. Anti-fungal treated mice had aggravated dextran sulphate sodium (DSS) -induced colitis, while antibiotic treated mice had less severe DSS-colitis. On further investigation, *Lactobacillus* growth was increased in anti-fungal fed mice, which in theory should have helped in causing less severe colitis, as *Lactobacillus* is known to promote gut health. It is hypothesized that the depletion of commensal intestinal fungi through the use of anti-fungals may trigger growth of pathogenic bacterial microbiota which in turn exacerbate colitis [43]. This illustrates a complex relationship between the mycobiome and microbiome and more can be done to fill the gaps in understanding of the interactions of the gut commensal organisms.

These studies underscore the influence of anti-bacterial and anti-fungal compounds on the intestinal flora in pathogen colonization. Many anti-bacterials are known to promote fungal growth and pathogenicity because they disrupt the microbiota and eliminate anaerobic bacteria in the gut which could have otherwise inhibited the fungi.

## 4. The Gut Mycobiome and Disease Susceptibility

Historically, because *Candida* species could be isolated using culture-dependant methods, it has been primarily implicated in various gut-related diseases such as inflammatory bowel diseases (IBD), Crohn’s disease (CD), ulcerative colitis (UC), and gut inflammation. (reviewed in [44]). As an extension, using culture-independent methods, researchers are uncovering the involvement of the other fungi in the gut mycobiome in relation to gastrointestinal diseases, and excellent reviews of the fungal microbiota and IBD can be found [45,46]. McKenzie et al. [47] found that CD patients had anti- *Saccharomyces cerevisiae* antibodies (ASCA), which implicated a role for the fungi in the inflammatory immune response in CD. Ott et al. [22] found increased fungal richness and diversity in CD patients compared to the controls. Similarly in another study, Li et al. [48] found increased fungal diversity in CD patients’ inflamed mucosa compared with non-inflamed mucosa. Liguori et al. [49] found altered bacterial and fungal microbiota—less operational taxonomic units (OTUs), less *Firmicutes*, more *Proteobacteria*, and larger fungal load in CD patients compared to healthy subjects. Recently, a study of the microbiota of 235 IBD patients revealed a large difference in alpha- and beta-diversity between healthy controls, UC patients, and CD patients, and although the beta-diversity clustering was weaker, the alpha-diversity of the fungal mycobiota was decreased in IBD patients compared with healthy controls [50]. Similar to Liguori et al. [49], Li et al., and Sokol et al. [48,50], found *C. albicans* in increased frequency in the IBD samples. This suggests that in IBD, the myco- and microbiome have a mutual influence on each other. Pointing to the role of the bacteria and microbial dysbiosis in IBD, a study that reviewed randomised controlled trials found significant effects of broad-spectrum antibiotics in the treatment of active CD and active UC [51]. Taken together, these studies suggest that bacterial dysbiosis in the gut, and the richness and diversity of the mycobiome, are important in IBD susceptibility.

The fungal mycobiota is sensitive and responds (directly or indirectly) to disease states and intervention, oftentimes iatrogenically through treatment. Qiu et al. [43] showed in mice that fungal compositions vary between DSS-induced colitis and normal states. In DSS-induced colitis mice, fungal biodiversity was narrowed as compared to normal controls in different gut segments [43]. In mice, a group found fungal dysbiosis induced by anti-fungal treatment caused selection of rarer, non-*Candida* fungi. This led to worsening of DSS-induced colitis and exacerbated allergic airway disease [52]. Conversely, another study found patients receiving antibiotics, resulting in antibiotic-associated diarrhoea (AAD), had disturbance to the indigenous microbiota preceding *Candida* overgrowth in the GI tract (reviewed in [53]). *Candida* species and *Geotrichum candidum* have been implicated in causation of IBS in patients who had received prior anti-bacterials, as these patients seemed to respond to nystatin, an anti-fungal preparation [54,55]. This suggests that IBS may be caused by a disruption in the gut bacteria induced by antimicrobials, which at an opportune moment permit the commensal mycobiota pathobionts to grow out of proportion.

In colorectal cancer, researchers found that mucosal mycobiota differed between adenoma size and disease stage [56]. Adenomas had less diversity and the order Saccharomycetales was significantly enriched in advanced adenoma biopsy samples. Conversely, in the adjacent rectal tissue samples, *Fusarium* and *Trichoderma* genera were significantly enriched compared to non-advanced adenoma tissues [56]. This suggests that in patients with advanced adenomas, the mycobiota may exhibit differences in association with disease stage. Though this study only compared between advanced adenoma and non-advanced adenomatous tissues, it illustrated differences in the mycobiota in different stages of colorectal adenoma size. Moving forward, more studies need to be done to determine the cause-effect relationship between the mycobiota and colorectal cancer.

From such dysbiosis studies, we can see that the microbiome and commensal fungi interaction is a balancing act; when the microbiome is disrupted, the normally commensal elements in the mycobiome might be unchecked and turn pathogenic.

## 5. Dietary Influences and Obesity

There have been few studies on the influence of diet on the fungal mycobiota. Many inhabitants of our gut microbiome originate from the food consumed. Foodborne fungi are found in many animal and plant-based foods, surviving the transit through the digestive system and possibly transiently colonizing the gut [11]. Hoffmann et al. [17] reported a strong link between consumed foods and fungal abundance in the gut. They found an inverse association between *Candida* (fungus) and *Bacteriodes* (bacteria). *Bacteriodes* was more abundant in individuals consuming high protein diet while *Candida* abundance was strongly associated with recent consumption of carbohydrates. They also observed notable fungal-bacterial associations, such as positive correlations between *Fusarium* (fungus), *Bryantella* (bacteria), and *Anaerostipes* (bacteria); and *Pichia* (fungus) and *Syntrophococcus* (bacteria) [17], though the significance of these associations in vivo are not known. β-glucan, a major component of the fungal cell wall, decreased fecal *E. coli* counts in animals [57], suggesting the metabolism of the fungal cell wall in the gut might influence the growth of *E. coli* and other bacteria. Such studies of dietary manipulation and influence on the mycobiome are at their infancy and we look forward to greater understanding of the dietary significance of these bacteria-fungi correlations.

Between non-obese and obese subjects, a study found there were no significant differences in fungal richness; however, biodiversity—especially with Zygomycota, was lower in the obese subjects as compared to the non-obese. The most abundant genera found in obese patients were *Mucor* and *Nakaseomyces*. In line with this finding, the relative abundance of *Mucor* increased after diet-induced weight loss in the obese subjects [19]. In addition there were correlations between High-density lipoprotein (HDL)-cholesterol and the relative abundance of class Eurotiomycetes, family Aspergillaceae and genus *Penicillium*, and negative correlations from the classes Saccharomycetes, Tremellomycetes, Cystobasidiomycetes and family Erythrobasidiaceae amongst other metabolic parameters studied [19]. It remains to be elucidated how such variations in the mycobiota may influence the body fat metabolism or vice versa.

## 6. The Mycobiome and Immunity

Researchers found that to achieve cecal colonization by *C. albicans* in mice, pre-treatment with antibiotics and immunosuppressants was more effective than treatment with either antibiotics or immunosuppressants alone [58]. This suggested that there was interaction between the microbiome and the host immune system in mediating gut colonization.

In the innate immune system, immune cells express pattern recognition receptors (PRRs) on their surface to recognize pathogen associated molecular patterns (PAMPs), which are conserved motifs found on pathogens (reviewed in [59]). Dectin-1 is one such PRR, classified as a C-type lectin receptor, which recognizes the fungal polysaccharide β-1,3 glucan motif found on the fungal cell walls, and mediates host immune response to these fungi (reviewed in [59]). In the mouse model of DSS-induced colitis, dectin-1 knock-out (KO) mice had more severe colitis compared to wild-type, and there was an increase in *Candida* and *Trichosporon*, which are opportunistic pathogens, and decrease in non-pathogenic *Saccharomyces* [60]. This suggests that commensal fungi affect the extent of the inflammation in mice. The fungi invaded the inflamed tissues in the dectin-1 KO mice but stayed in the lumen of the wild type mice. Increased IL-17, IFN-γ, and TNF-α were detected in the dectin-1 KO mice colons. When given fluconazole, dectin-1 KO mice had milder symptoms and less weight loss due to colitis. This suggests that dectin-1 plays a role in preventing invasive disease. The authors also identified humans with dectin-1 polymorphism who had more severe, medically refractory ulcerative colitis [60], suggesting that the polymorphism does not make the patients susceptible to colitis per se, but contributes to the aggravation of colitis. The observation that the fungi contributed to the aggravation of inflammatory response in this model pointed to dectin-1 being part of the immune mechanism to keep commensal fungi in check. In another study, a dectin-1 SNP associated in UC was negatively correlated with relative abundance of *Malassezia sympodialis*, although it did not pass the threshold of significance [50]. These reports suggest gut commensal fungi interaction with the innate or mucosal immune system which remains to be further elucidated.

The gut mycobiota may influence immune responses in other parts of the body, such as in extra-gastrointestinal organs. A study by Wheeler et al. [50] had demonstrated in mice with DSS-induced colitis that oral anti-fungals leading to altered gut mycobiota resulted in exacerbation of allergic airway disease. In addition, another study by McAleer et al. [61] also demonstrated the influence of gut microbiota on the pulmonary immune response. Mice fed with vancomycin in their drinking water for one month had significantly decreased IL-17 and IL-22 levels and increased IL-4 levels in their lungs following *A. fumigatus* oropharyngeal challenge. This points to how vancomycin-sensitive gut commensals may modulate Th-17 and Th-2 responses against fungi in the lungs.

## 7. The Role of Extracellular Substances Produced by the Commensal Microbiota in the Gut in Preventing Pathogenicity and Maintaining the Myco-Microbiome Balance

The myco- and microbiome may be perceived as being in a state of homeostasis. One of the ways the bacteria in the microbiome keep the mycobiome in check is the production of extracellular substances that inhibit the growth or yeast-to-hyphae transition of pathobionts, such as *C. albicans*. Clinically, the use of these substances to prevent pathogenicity may potentially offer an alternative to anti-fungal drugs for prophylactic and therapeutic uses, as anti-fungal use skews the mycobiota and selects for drug-resistant strains. The commensal microbiota is known to produce a wide range of small molecules which have inhibitory effects to pathogens, such as short chain fatty acids (SCFA), medium-chain fatty acids (MCFA), secondary bile acids, bacteriocins, and antimicrobial peptides, as reviewed in [62,63,64,65]. Acetic, propionic, and butyric acid have been demonstrated to induce transcriptional changes in *C. albicans* [66], and butyric acid was found to inhibit yeast-hyphal (Y-H) transition (reviewed in [67]). Similarly, the antimicrobial peptide LL-37 is believed to be directly active against *C. albicans* [68] and indirectly through commensal anaerobic-linked protection against *C. albicans* gut colonization. This has been implicated in reducing gastrointestinal colonization by *C. albicans* [69]. The microbiota also produces gases from fermentation, such as hydrogen, methane, and hydrogen sulphide (reviewed in [64]). The fungal gut flora may produce small molecules like farnesol and fusel alcohols which could regulate on their own Y-H transition. Similarly, capric acid from *S. boulardii*, a probiotic yeast, could influence Y-H transition, adhesion, and biofilm formation (reviewed in [67]). In turn, ethanol from *S. cerevisiae* has been shown to stimulate the growth of *Acinetobacter* in vitro [70], and similar yeasts are found in the gut mycobiome. Fungi are known to produce a diverse array of secondary metabolites and more studies can be done to elucidate the contribution of the mycobiome to the substances produced in the gut in health and disease.

## 8. Conclusions

In the future, there will be more studies looking at the mutualism between the myco- and microbiome, and the consequences of dysbiosis—be it through lifestyle, disease, or iatrogenically through treatment. It is clear that dysbiosis of the fungal mycobiome can cause as much havoc in our body as dysbiosis of the bacteria microbiome. Most of the findings to date on the microbiome and mycobiome remain largely descriptive or associative, and originating primarily from the gut. We anticipate further inroads into understanding bacteria-fungi interaction moving beyond GI tract into the airway, and more importantly, attempts at synergizing metagenomics with functional studies to elucidate the mechanisms of diseases. Nonetheless, the eventual goal is to determine how we may harness the yields from the above endeavours to derive novel therapeutics to improve outcomes of disease, both in infectious diseases and beyond.
